# Nano-Assembly of Quisinostat and Biodegradable Macromolecular Carrier Results in Supramolecular Complexes with Slow-Release Capabilities

**DOI:** 10.3390/pharmaceutics13111834

**Published:** 2021-11-02

**Authors:** Ananda Chowdhury, Alexander Marin, David J. Weber, Alexander K. Andrianov

**Affiliations:** 1 Institute for Bioscience and Biotechnology Research, University of Maryland, Rockville, MD 20850, USA; ananda.d22@gmail.com (A.C.); amarin1@umd.edu (A.M.); DWeber@som.umaryland.edu (D.J.W.); 2 Department of Biochemistry and Molecular Biology, University of Maryland School of Medicine, 108 N. Greene St., Baltimore, MD 21201, USA; 3 Center for Biomolecular Therapeutics (CBT), Baltimore, MD 21201, USA; 4 Marlene and Stewart Greenebaum Cancer Center, University of Maryland School of Medicine, Baltimore, MD 21201, USA

**Keywords:** quisinostat, polyphosphazenes, PEGylation, slow-release, histone deacetylase inhibitors

## Abstract

Self-assembly of ionically charged small molecule drugs with water-soluble biodegradable polyelectrolytes into nano-scale complexes can potentially offer a novel and attractive approach to improving drug solubility and prolonging its half-life. Nanoassemblies of quisinostat with water-soluble PEGylated anionic polyphosphazene were prepared by gradient-driven escape of solvent resulting in the reduction of solvent quality for a small molecule drug. A study of binding, analysis of composition, stability, and release profiles was conducted using asymmetric flow field flow fractionation (AF4) and dynamic light scattering (DLS) spectroscopy. Potency assays were performed with WM115 human melanoma and A549 human lung cancer cell lines. The resulting nano-complexes contained up to 100 drug molecules per macromolecular chain and displayed excellent water-solubility and improved hemocompatibility when compared to co-solvent-based drug formulations. Quisinostat release time (complex dissociation) at near physiological conditions in vitro varied from 5 to 14 days depending on initial drug loading. Multimeric complexes displayed dose-dependent potency in cell-based assays and the results were analyzed as a function of complex concentration, as well as total content of drug in the system. The proposed self-assembly process may present a simple alternative to more sophisticated delivery modalities, namely chemically conjugated prodrug systems and nanoencapsulation-based formulations.

## 1. Introduction

Histone deacetylase inhibitors (HDACis) represent a class of promising chemotherapeutic agents [1] with a number of these compounds, such as belinostat, chidamide, panobinostat, romidepsin, and vorinostat, already approved for clinical use under different jurisdictions [2]. HDACIs have been shown to induce differentiation, cell-cycle arrest, and apoptosis in many cancer cell lines [2]. However, clinical results with HDACis as monotherapies have been either modest or disappointing and these drugs are currently used in combination with other therapies [2,3,4]. These limitations are reportedly imposed by low bioavailability and short half-life of drugs [5,6], as well as a number of class- and agent-specific serious or severe adverse effects, notably myelosuppression and cardiac effects, associated with their clinical use [2]. Quisinostat is a second generation HDACi, which has already showed improved pharmacodynamic effects in vivo and demonstrated superior antitumoral efficacy compared to other analogs [7,8,9,10,11,12]. Nevertheless, the search for formulation and delivery approaches that can further prolong drug exposure, minimize drug toxicity to normal tissues, and improve therapeutic index continues [2,6,13,14].

To date most of the research activity in the field has been focused on efforts of encapsulating quisinostat into various drug carriers. To that end, various macromolecular systems have been explored, such as nanoparticles on the basis of poly(lactide)-block-poly(ethylene glycol) [15], poly (lactide-co-glycolide)-lecithin-poly(ethylene glycol) [6], and polysaccharide [16], as well as matrices composed of β-cyclodextrin-poly (β-amino ester) networks [17] and bioerodible radiopaque hydrogels [14]. Despite offering multiple distinct advantages, all of these methods usually involve sophisticated chemical and formulation approaches and require multi-step production processes.

An alternative methodology to the delivery of small cationic drugs, which display inferior pharmacokinetic and pharmacodynamic (PK/PD) profiles, was proposed recently [18]. The approach is based on an ionic coupling of cationic drug to a negatively charged biodegradable polyelectrolyte. Although the strength of association between polyelectrolyte and its counterions has typically been considered to be insufficient to withstand physiological conditions, the in vitro and in vivo feasibility of such a concept was recently demonstrated for supramolecular assemblies between macromolecular polyphosphazene immunoadjuvants [19] and a small molecule immunomodulator—resiquimod/R848 [18,20]. We recently introduced anionic polyphosphazenes with biodegradable backbone containing graft poly(ethylene glycol) PEG chains (PPEGs), which were characterized by improved water-solubility and stability to aggregation [21]. These polymers were explored as non-covalent PEGylation agents for extending half-life of proteins [21] and intracellular delivery of peptide and protein cargo, and were shown to be effective in facilitating cellular uptake of protein cargo and non-toxic to cells [22]. Applications of such PEGylated macromolecules to the delivery of HDACis can offer a simple single-step formulation alternative to more sophisticated and labor intense methodologies.

Here, we report a single-step approach to the preparation of nano-scale water-soluble supramolecular complexes of quisinostat via its spontaneously self-assembly with biodegradable PEGylated macromolecular carrier (PPEG). The resulting multimeric complexes contain up to 100 quisinostat molecules per polymer chain, display extended drug release profiles under near physiological conditions, and show improved hemocompatibility in the hemolysis assay compared to drug alone. Assessment of these nano-scale assemblies in cellular assays with WM115 melanoma cell and A549 lung cancer cell lines confirms their potency in solvent free formulations, as well as their freeze-thaw and lyophilization stability.

## 2. Materials and Methods

Materials. Quisinostat dihydrochloride, 97%; dimethyl sulfoxide, DMSO (Sigma-Aldrich, St. Louis, MO, USA), resazurin cell viability kit (Cell Signaling Technology, Danvers, MA, USA); porcine red blood cells, RBCs (Innovative Technology Inc., Novi, MI, USA); phosphate buffered saline pH 7.4, PBS (Life Technologies, Carlsbad, CA, USA); and cell culture media and regents (Thermo Fisher Scientific, Waltham, MA, USA) were used as received. Poly[di-(carboxylatoethylphenoxy)phosphazene]-graft-poly(ethylene glycol) (PPEG) containing 17% (mol) of 5 kDa PEG groups, molecular weight 150 kDa, was synthesized as described previously [21].

Preparation of Quisinostat Loaded Polymer Complexes. Stock solutions of quisinostat and PPEG were prepared in DMSO-deionized (DI) water solvent mixture (5% (*v*/*v*) and 95% (*v*/*v*), correspondingly) and were filtered using 0.22 μm Millex syringe filters (EMD Millipore, Billerica, MA, USA). A series of quisinostat—polymer formulations were prepared by mixing stock solutions of components at various volumetric ratios, which provided for molar excess of drug compared to PPEG. The formation of complexes was driven by removal of DMSO from formulations, which was achieved by creating concentration gradient against DI water using SpectraPor regenerated cellulose membrane with 50 kDa molecular weight cutoff (Repligen, Boston, MA, USA). The procedure was conducted for 7 h at ambient temperature and was monitored by DLS to ensure the absence of aggregation in the system. Diffusion dialysis of formulations containing various concentrations of quisinostat in the absence of PPEG, PPEG alone, and DMSO-water solvent mixture was carried out using the identical set-up and formulations were monitored by UV spectroscopy to establish kinetics of release for formulation components.

Physico-Chemical Characterization of Complexes. Asymmetric Flow Field Flow Fractionation (AF4) analysis of the complexes was carried out using AF2000 MT (Postnova Analytics GmbH, Landsberg, Germany). Malvern Zetasizer Nano series (Malvern Instruments Ltd., Worcestershire, UK) was employed for DLS measurements. Composition of complexes was evaluated by first determining molar attenuation (extinction) coefficient of quisinostat in water-DMSO mixture and analyzing complexes by either UV spectrophotometry or AF4 (increase in complex peak area compared to polymer peak). Release experiments were conducted in Franz diffusion cells using 10-fold excess of PBS to maintain sink conditions (10 mL, PermeGear, Inc., Hellertown, PA, USA) equipped with regenerated cellulose membrane (30 KDa cut off) in PBS, pH 7.4.

Hemolytic Activity Assay. The hemocompatibility of complexes, PPEG, and quisinostat was evaluated using hemolysis test with porcine red blood cells, RBCs (Innovative Technology Inc., Novi, MI, USA) [23,24]. Briefly, 50 μL of fresh RBCs as a 1% suspension in PBS was added to 950 μL of test formulation and incubated at 37 °C for 60 min. Cells were then centrifuged at 14,000 rpm, and the absorbance of the supernatant was measured at 541 nm. For 100% hemolysis, RBCs were suspended in distilled water. All experiments were done in triplicates.

Evaluation of Quisinostat-PPEG Complexes in Cellular Assays. The cytotoxicity of Quisinostat loaded PPEG formulations and individual controls was evaluated in WM115 melanoma cell (CRL-1675) line and A549 (CCL-185) lung cancer cell line (ATCC, Manassas, VA, USA). Briefly, the WM115 and A549 cells were grown and maintained in Minimum Essential Media (1× MEM) and F-12K media, respectively, supplemented with 10% Fetal Bovine Serum and 5% Penn-Strep. A total of 180 µL of media containing 100,000 cells/mL were seeded in each well of sterile tissue culture grade 96-well plate. Treatments and controls were formulated at 10× concentration in cell culture media and further diluted to prepare the dose concentrations (pre-treatment stocks). A total of 20 µL pre-treatment stocks were added to each well and incubated at 37 °C for 72 h. Cell viability of treatment wells and no-treatment controls was determined at 72 h using resazurin reduction fluorometric assay [25,26]. The fluorescence signal of live cells was recorded using a Spectra Max plate reader (Molecular Devices, LLC, San Jose, CA, USA) using Excitation/Emission wavelengths of 550 and 605 nm, respectively.

Statistical Analysis. The dose response viability curves were generated using percentage viability data of the respective cell lines (*n* = 3) plotted against the log_10_ molar concentrations of quisinostat or complex using GraphPad Prism software. Curves were plotted using non-linear regression using a response vs. log (inhibitor) three parameter model. Points represent the mean values with errors indicating standard deviation. Respective IC_50_ values, 95% confidence intervals of IC_50_ values, and goodness of fit (R^2^) of individual curves are shown in Table A1, Table A2, Table A3 and Table A4.

Stability Studies. The test article was either lyophilized and then reconstituted in DI water or underwent freezing at −20 °C and thawed at ambient temperature. It was then assessed for potency in WM115 melanoma cell viability assay and analyzed for potential changes in dimensions and aggregation by DLS.

## 3. Results

### 3.1. Solvent Gradient Driven Nano-Assembly of Quisinostat—Polyphosphazene Complexes

**Figure 1:**
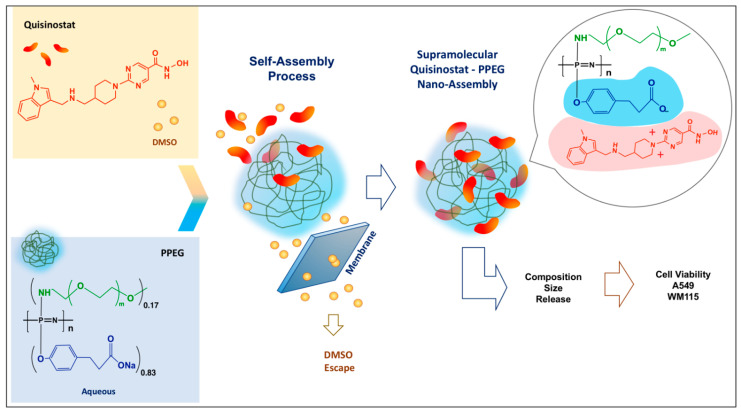
Schematic diagram of quisinostat-polyphosphazene self-assembly process and study workflow.

**Figure A1:**
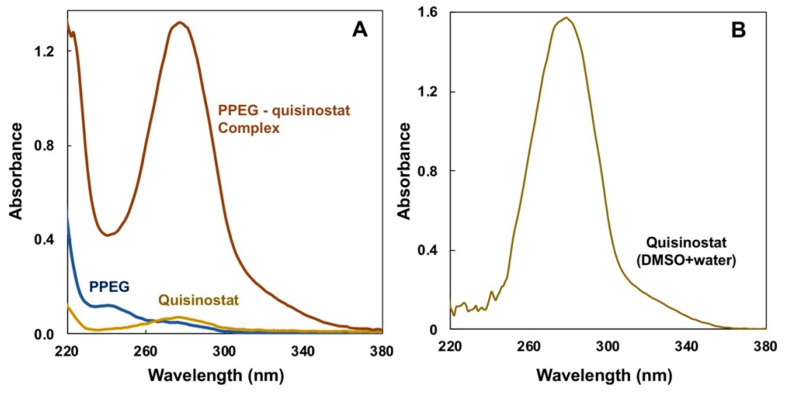
UV profiles of (**A**) PPEG–quisinostat complex after dialysis-driven self-assembly compared with the components (PCPP-PEG and quisinostat) dialyzed individually (results for complex with 15:1 quisinostat-to-polymer ratio are shown) and (**B**) quisinostat in DMSO-water mixture.

The process of self-assembly of quisinostat—a cationic small molecule drug (“counterion”) and anionic PEGylated polyphosphazene—PPEG (“polyelectrolyte”) was driven by a gradual removal of DMSO—a cosolvent needed for maintaining solubility of the drug [15,27,28,29]. This process, which can be described as a “counterion condensation” [30,31,32,33] was conducted in the dialysis cell under conditions allowing DMSO and any unbound quisinostat to gradually escape from the formulation via creation of concentration gradient against DI water using the semipermeable membrane (Figure 1). Care was taken to avoid aggregation—polymer “chain collapse”, as monitored by DLS. The duration of self-assembly procedure was determined in dialysis studies with individual formulation components and satisfied the following two criteria. First, the time allowed for a complete release of DMSO through the membrane as monitored by UV measurements. Second, when the dialysis was conducted in the absence of PPEG carrier, the time was sufficient for quisinostat to either escape the cell or precipitate on the surface of the membrane. UV-analysis of these dialyzed drug solutions in the absence of PPEG, which originally contained same amounts of quisinostat as in polymer formulations, confirmed practical absence of drug (Appendix A, Figure A1A). This was in a contrast with PPEG-based formulations demonstrating strong absorbance in the vicinity of 280 nm and confirming the presence of quisinostat (Appendix A, Figure A1B).

### 3.2. Quisinostat Associates with PPEG in a Dose Dependent Manner

**Figure 2:**
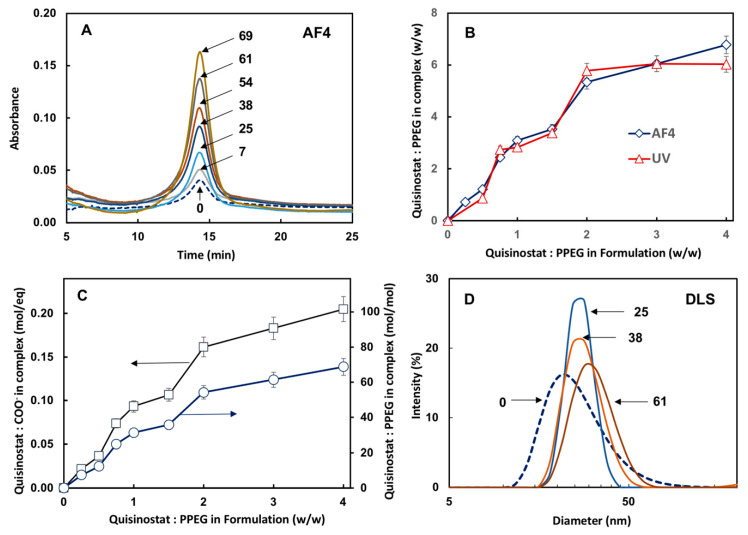
(**A**) AF4 fractograms of PPEG and quisinostat-PPEG complexes showing growing macromolecular peak area as the content of drug increases; (**B**) comparison of polymer composition as measured by AF4 and UV-spectroscopy; (**C**) composition of complex expressed as degree of neutralization of anionic group on PEPG and drug-to-polymer molar ratio as a function of quisinostat-to-PPEG ratio in the original formulation; (**D**) DLS profiles of quisinostat-PPEG complexes (numbers in panels (**A**,**D**) indicate quisinostat-to-PPEG molar ratio in the complex).

**Figure A2:**
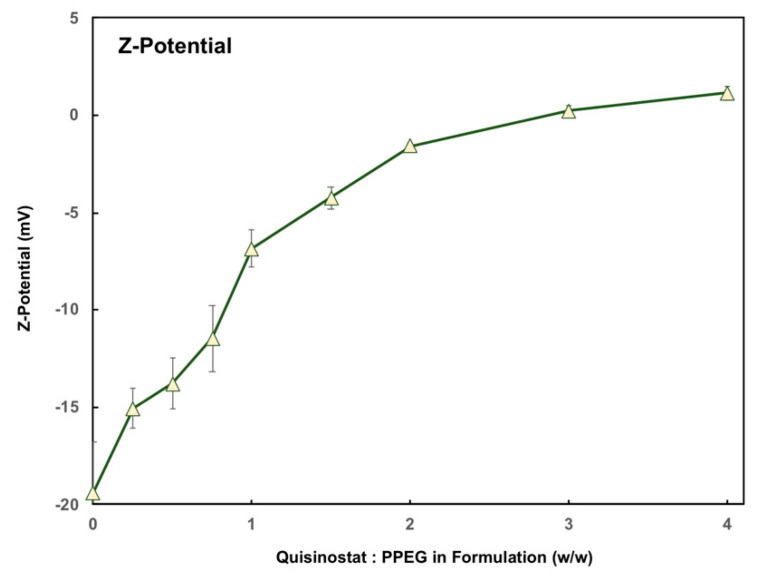
Z-potential measurements for PPEG–quisinostat formulations (*n* = 3; error bars represent standard deviation).

First, the comparative analysis of quisinostat-PPEG composition was conducted using AF4, which allows separation of analytes on the basis of their molecular or supramolecular dimensions [34], and UV spectrophotometry methods. Since the AF4 method does not provide for the reliable analysis of small molecule drug, which generally escapes through the semipermeable membrane before reaching the detector, the analysis of formulations was focused on the macromolecular (PPEG) peak—14 min retention time (Figure 2A). The results clearly show a quisinostat dose dependent increase in the peak area indicating association of drug with the polymer. The drug loading was evaluated on the basis of AF4 and UV-analysis of quisinostat-complexes using molar attenuation coefficient determined for water-DMSO system and demonstrated excellent correlation between both methods (Figure 2B). The dependence of quisinostat loading, calculated as a number of drug molecules carried by a single PPEG chain and the ratio of quisinostat-to-carboxylic acid groups, on the composition of initial formulations are shown in Figure 2C. Z-potential measurements show gradual increase from electronegativity (−20 mV for the polymer) to electroneutrality for highly loaded complexes (Figure A2), which is consistent with neutralization of PPEG with quisinostat counterions. Finally, dynamic light scattering results show the absence of aggregation in formulations and a slight shift towards larger molecular dimensions of supramolecular complexes (Figure 2D).

### 3.3. Drug Release Characteristics and Hemocompatibility of Complexes

**Figure 3:**
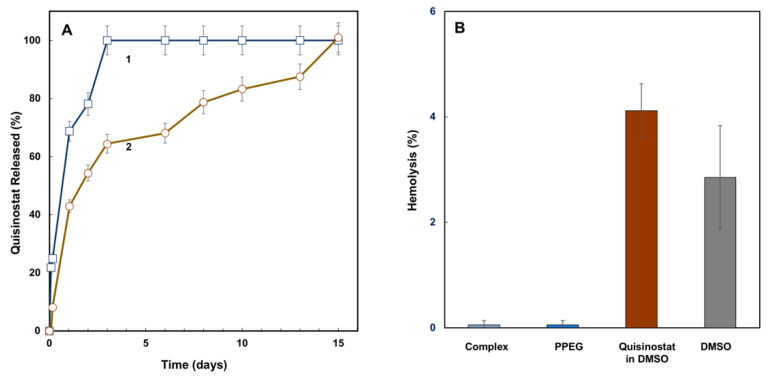
(**A**) Kinetics of quisinostat release from complex expressed as a percent of initial load of drug for samples with quisinostat to PPEG molar ratio in the complex of 5 (1) and 38 (2) (PBS, pH 7.4; Franz diffusion cell); (**B**) hemolytic activity of quisinostat-PPEG complex (activity for 38 drug-to-polymer molar ratio, other complexes are similar), PPEG solution of quisinostat in 10% (*v*/*v*) DMSO and 90% (*v*/*v*) PBS mixture (RBCs, 37 °C, 1 h, n = 3, mean values reported, error bars represent standard deviations).

The release of quisinostat from complexes was studied in near physiological environment under sink conditions (PBS, pH 7.4). Two complexes containing medium and low drug load (28 and 5 quisinostat-to-PPEG molar ratios) were chosen for these studies. The rationale for this selection was based on the well-known inverse relationship between of dissociation constant and degree of polyelectrolyte dissociation [33,35]. Accordingly, complexes with lower load of positively charged quisinostat counterions (higher negative charge of the complex) are more likely to resist undesirable “burst” release of drug due to stronger electrostatic interactions. Both complexes displayed slow-release profiles with complexes dissociating and counterion being released within several days (Figure 3A). While low drug load complex was characterized a relatively short period of release—approximately two days—the medium load formulation retained quisinostat for up to two weeks. Hemocompatibility of quisinostat-PPEG formulations was evaluated in hemolysis assay [23,24] using porcine red blood cells, RBCs (Figure 3B). Both complex and PPEG showed significantly reduced hemolytic activity compared to drug formulation in DMSO-PBS solvent mixture and the respective carrier—DMSO-PBS.

### 3.4. Potency of Quisinostat-PPEG Complexes in Cell-Based Assays

**Figure 4:**
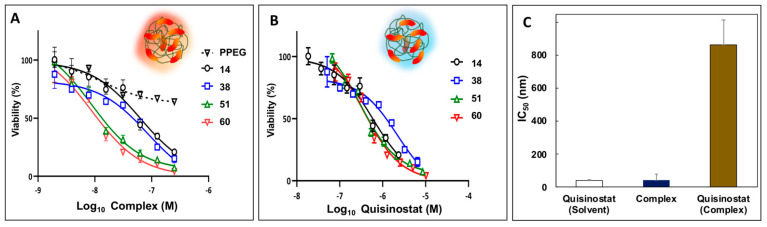
Potency of quisinostat-PPEG complexes in A549 lung cancer cell viability experiments: (**A**,**B**) Dose–cell viability curves for quisinostat-PPEG complexes and PPEG plotted as a function of complex/polymer (**A**) or quisinostat (**B**) concentrations (numbers indicate quisinostat to polymer ratios in the complexes (mol/mol)); (**C**) half maximal inhibitory concentrations (IC_50_) calculated relevant to concentrations of quisinostat formulated in DMSO-water mixture (“quisinostat-solvent”), concentration of entire complex containing multiple quisinostat molecules (“complex”—panel (**A**) data, average), and individual quisinostat molecules in a complex—“quisinostat-complex”—from panel (**B**) data (72 h; n = 3; points represent the mean values with errors indicating standard deviation; respective IC_50_ values, 95% confidence intervals of IC_50_ values, and goodness of fit (R^2^) of individual curves are shown in Appendix B, Table A1 and Table A2).

It has been well documented that, in vitro, quisinostat exerts strong anti-proliferative activity in a nanomolar range against non-small cell lung cancer cell lines and A549 human lung cancer cells in particular [9,10]. Therefore, the potency of quisinostat-PPEG complexes was first evaluated in assays with A549 human lung cancer cells. All quisinostat-PPEG complexes, but not PPEG alone, were able to significantly inhibit A549 cell proliferation in dose- and time-dependent manners. The results of experiments after 72 h treatment were presented in two different modes. First, dose-response relationships were displayed relevant to concentration of the entire complex, which contained multiple drug copies (Figure 4A). This type of analysis assumes that the activity of formulation is inherently associated with that of the entire complex, but not with individual quisinostat molecules. Second, the same results were presented as a function of concentration of individual quisinostat molecules in the system (Figure 4B), which may be more informative if complex dissociates during the analysis or multimericity plays a significant role. Half maximal inhibitory concentration (IC_50_) calculated on the basis of the entire complex was in the 9–84 nM range depending on the loading (Appendix B, Table A1), and in agreement with the value reported in the literature for solvent based quisinostat formulations [9] (Figure 4C, columns designated as “complex” (from Figure 4A data, average value) and “quisinostat (solvent)”, respectively). However, the same value obtained relevant to concentration of individual drug molecules in the system was approximately an order of magnitude higher, as shown in Figure 4C, column “quisinostat–complex” (from Figure 4B data (average) and Table A2, Appendix B).

**Figure 5:**
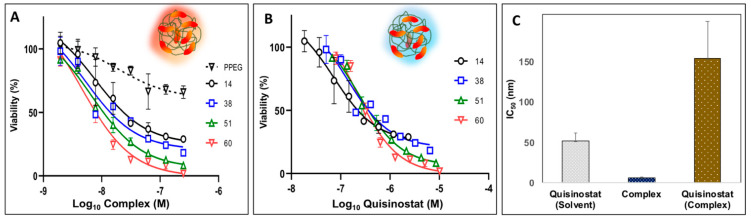
Potency of quisinostat-PPEG complexes in WM115 human melanoma cell viability experiments: (**A**,**B**) Dose–cell viability curves for quisinostat-PPEG complexes and PPEG plotted as a function of complex/polymer (**A**) or quisinostat (**B**) concentrations (numbers indicate quisinostat to polymer ratios in the complexes (mol/mol)); (**C**) half maximal inhibitory concentrations (IC_50_) calculated relevant to concentrations of quisinostat formulated in DMSO-water mixture (“quisinostat-solvent”), concentration of entire complex containing multiple quisinostat molecules (“complex”—panel (**A**) data, average), and individual quisinostat molecules in a complex—“quisinostat-complex”—from panel (**B**) data (72 h, n = 3; points represent the mean values with errors indicating standard deviation; respective IC_50_ values, 95% confidence intervals of IC_50_ values, and goodness of fit (R^2^) of individual curves are shown in Appendix B, Table A3 and Table A4).

**Figure A3:**
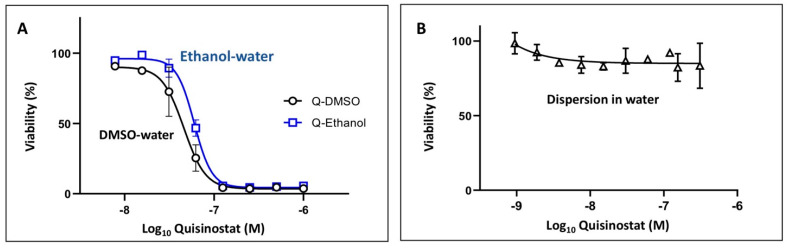
Representative dose–cell viability curves for quisinostat (**A**) dissolved in DMSO-water or ethanol-water mixtures and (**B**) dispersed in water (WM115 human melanoma cell line; 72 h, *n* = 3; points represent the mean values with errors indicating standard deviation).

Next, potency of quisinostat-PPEG complexes was evaluated in WM115 human melanoma cell line, which is frequently used to evaluate both macromolecular drug delivery systems and HDAC inhibitors [36,37]. As with A549 cells, quisinostat-PPEG complexes of different loadings were effective in decreasing the viability of WM115 cells in a dose-dependent manner. Similar to A549 cell experiments data, dose–viability curves display dependence on both complex and quisinostat concentrations (Figure 5A,B). Once again, IC_50_ calculated on the concentration of the entire complex was approximately five-fold lower than the same value obtained on the basis of individual quisinostat molecules in the complex (Figure 5C). However, in contrast with A549 data, the IC_50_ value for the entire multimeric complex was also lower than that for quisinostat in standard quisinostat vehicles -DMSO-water or ethanol-water mixtures [27,28]—4.5–7.6 vs. 52 nM (Appendix B, Table A3 and Table A4 and Figure A3A). For comparison, IC_50_ of doxorubicin in melanoma cell lines was reported in a micromolar range—5 µM [38]. It has to be also noted that no cytotoxicity was observed for quisinostat dispersed in aqueous solution without cosolvent (Appendix B, Figure A3B) or for DMSO formulated drug (no PPEG), which underwent dialysis under same conditions as polymer formulations for both cell lines.

### 3.5. Complexes Demonstrate Stability in Freeze-Thaw and Lyophilization Stability Tests

**Figure 6:**
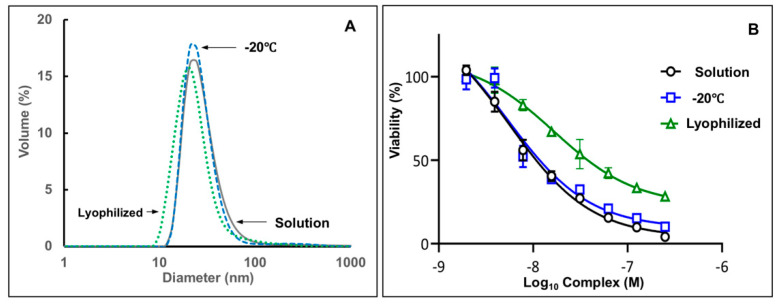
(**A**) DLS profiles and (**B**) dose-potency relationship in cell-based viability assay for quisinostat-PPEG complexes in solution, after freeze/thaw cycle (−20 °C) and lyophilization (complex composition: 51:1 quisinostat-to-PPEG; solution in 25 mM phosphate buffer; viability experiments: WM115 Melanoma Cells; *n* = 3; points represent the mean values with errors indicating standard deviation).

Stability of complexes under freeze/thaw and lyophilization stresses was assessed by monitoring potency in cell-based assay and size distribution analysis of formulations by DLS in phosphate buffer. A high quisinostat load complex (51 drug-to-polymer molar ratio) was selected for the study as potentially less resistant to aggregation due to high charge neutralization degree [39]. No change in particle size distribution (Figure 6A) or potency of complex assessed in WM115 melanoma cell viability assay (Figure 6B) was detected after freeze-thaw cycle, which was conducted by exposing formulation to −20 °C and thawing the sample at ambient temperature. The complex, which was lyophilized and then reconstituted in DI water, displayed minor shift towards smaller sizes without change in the dispersity index. These observations, which suggest slight compaction of macromolecular chain of the complex, were also accompanied by minor loss of potency—IC_50_: 17 nM vs. 6 nM for the sample before lyophilization.

## 4. Discussion

Physico-chemical aspects of self-assembly in quisinostat-PPEG system and an underlying design of polyphosphazene carrier can be discussed in the framework of the following considerations. Quisinostat is a positively charged small molecule drug, which can be potentially associated with negatively charged water-soluble macromolecules as a counterion. The strength of counterion attraction to polyelectrolytes is largely defined by a fine interplay between electrostatic interactions and the loss of translational entropy by counterions due to their proximity to polyelectrolytes [33,40,41]. In aqueous formulations the latter factor is typically prevalent and most polyelectrolytes release counterions into solution. Therefore, it was largely assumed that such constructs may be physiologically unstable [42,43,44]. However, hydrophobic organic counterions are expected to minimize unfavorable contacts with water molecules, which can lead to a desirable counterion “condensation” and physiologically stable drug-carrier complexes [43,45,46]. Unfortunately, this phenomenon can also cause potential collapse of polymer chain and undesirable phase separation [31,32,40,47]. In order to maintain water-solubility and stability of the system, graft-PEG chains were introduced in the structure of anionic polyphosphazene.

The process of quisinostat-PPEG self-assembly (Figure 1) was largely driven by the removal of DMSO resulting in a decrease of the solvent quality, the method which is known to induce spontaneous condensation of counterions [40]. Such gradual decrease in a content of a good solvent was achieved by creating concentration gradient in a membrane separation procedure and was not accompanied by phase separation in the system. In fact, DLS studies indicated only minimal increase in the z-average hydrodynamic diameter of the resulting formulation without significant changes in size distribution or any signs of aggregation (Figure 2D). The proof of quisinostat association with PPEG in the resulting formulation was achieved by AF4 method (Figure 2A). This technique is capable of size-dependent analysis of macromolecules using general methodology and equipment typical for high performance liquid chromatography. However, in contrast to chromatography, the separation is achieved by forcing the analyte against the semipermeable membrane by a perpendicular flow of the mobile phase [34]. Since the membrane is selected to be permeable to small molecules—quisinostat escapes through the membrane before reaching the detector—the detection of drug is only possible if it is bound to PPEG and its association with the polymer can withstand strong flux of phosphate buffer ions, which was used as a mobile phase. AF4 fractogram of formulations containing various doses of quisinostat shows dose dependent increase in the macromolecular (14 min) peak of the analyte (Figure 2A). This unambiguously proves formation of quisinostat-PPEG complexes and their ability to resist competitive exchange reactions with a large excess of sodium and potassium ions of the buffer flowing in the perpendicular direction to the analyte. Moreover, comparison of results generated by AF4 method, which can specifically detect polymer bound quisinostat, with data obtained by undiscriminating UV analysis of the formulation revealed good correlation (Figure 2B). This confirms the absence of unbound quisinostat in the formulation. The composition of multimeric complexes can be controlled through drug-to-polymer ratio in the initial formulation with a maximum load of quisinostat achieving approximately 100 molecules per polyphosphazene chain (Figure 2C).

Quisinostat release experiments demonstrated the ability of the complex to retain its drug cargo at near physiological conditions for up to two weeks (Figure 3A). This appears to be superior to results obtained for polylactide based nanoparticles (1–5 days) [6,15], and can open substantial opportunities for the preparation of slow-release formulations and potential improvement of therapeutic index. The time of half and total release was dependent on drug loading and was longer for high load formulation. All formulations displayed improved hemocompatibility compared to DMSO formulated drug (Figure 3B).

Quisinostat demonstrates high antitumoral efficacy when dissolved in DMSO-water mixture with selectivity index of 9 [48]. In vitro anti-proliferative activity of quisinostat-PPEG complexes in the absence of co-solvent was demonstrated using two cell lines—A549 lung cancer cells and WM115 human melanoma cells. The selection of these cell lines was made due to prior reports on evaluation of quisinostat with A549 lung cancer cells [9,10] and proven utility of WM115 human melanoma cells for the assessment of macromolecular drug delivery vehicles [36,37]. The results of these studies can be reviewed considering two outermost mechanistic modalities: potential activity of the entire multimeric complex as a single entity (dose-effect relationship analyzed vs. concentration of the complex—Figure 4A and Figure 5B) and, more traditionally, assuming that quisinostat is active once its molecules are detached from the polymer, analysis of dose-effect curves plotted against concentration of a drug (Figure 4B and Figure 5B). The first hypothesis may be considered taking into account established ability of PEGylated polyphosphazenes to facilitate uptake and intracellular delivery of its cargo, which has been demonstrated mainly for delivery of proteins and peptides [22,49,50]. The second assumption can be supported by the ongoing release of quisinostat under the conditions similar to those of viability experiments (Figure 3A,B). Comparison of IC_50_ values calculated in relevance to concentrations of ‘quisinostat’ molecules (either in solvent or complex—left and right column in Figure 4C and Figure 5C, respectively) and entire ‘complex’ (middle column in Figure 4C and Figure 5C) suggests that the latter values show better correlation with data for solvent-based formulations of drug. These results indicate that potency of macromolecular formulations is mainly defined by the entire complex, which is also supported by the observation that IC_50_ calculated on the basis of individual quisinostat molecules is approximately one order of magnitude higher than those calculated for the entire complex (Table A1, Table A2, Table A3 and Table A4). This apparent ‘loss of activity’ may be potentially explained by the multimeric nature of the complex; quisinostat molecules bound to the same polymer chain are unlikely to simultaneously interact with multiple cells due to a dramatic difference in size (nanoscale dimensions of polymer complex vs. micron scale dimensions of cells). Nevertheless, the importance of this observation needs to be explored in future experiments and the effect of complex dissociation and slow release of quisinostat cannot be disregarded.

Freeze-thaw and lyophilization stability of pharmaceutics are important factors affecting their feasibility for further development [51,52]. They can determine stability of future macromolecular product under various storage conditions, its reliance on cold-chain supply, and possibility of solid-state distribution with reconstitution before administration. Quisinostat-PPEG complexes appear to be stable in a freeze-thaw cycle and undergo only minimal change during lyophilization (Figure 6), which may indicate some subtle ion-induced conformational changes in the polyelectrolyte complex. Nevertheless, the potency of the complex is maintained in the original nanomolar range and can be further optimized by selecting a more appropriate ionic environment.

## 5. Conclusions

The results presented in this paper demonstrate that small cationic drugs, such as quisinostat, can be successfully assembled on a biodegradable macromolecular carrier through a single-step process, which involves gradient-driven reduction in solvent quality of the formulation. The resulting nano-scale supramolecular assemblies display excellent water-solubility and multimericity, retaining multiple molecules of quisinostat for as long as two weeks. These slow-dissociating supramolecular complexes also display superior hemocompatibility compared to common cosolvent-based aqueous formulations of quisinostat. Potency of quisinostat-polymer complexes was validated in cell-based assays using WM115 human melanoma and A549 human lung cancer cell lines. The study highlights the importance of often neglected ability of polyelectrolyte carriers to resist ion exchange of hydrophobic counterions under physiological conditions—the property is yet to be explored by pharmaceutical scientists for practical applications. The approach can be potentially extended to a larger scope of poorly soluble small molecule drugs, for which the development is hindered by inferior bioavailability, poor water-solubility, or unacceptable toxicity. It provides a simple alternative to more sophisticated methodologies for creating prodrug and slow-release formulations, such as covalent conjugation or nanoencapsulation.

## Data Availability

All available data included in the manuscript.

## Appendix A

Figure A1A compares UV profiles of three aqueous formulations after undergoing the dialysis driven self-assembly process: PPEG with quisinostat, quisinostat, and PPEG. As seen from the figure, polymer-based drug formulation shows strong UV absorbance in the vicinity of 280 nm, which is typical for the UV profile of quisinostat in DMSO-water mixture (Figure A1B). In contrast, neither PPEG nor quisinostat formulation, which underwent dialysis procedure, show absorbance at this wavelength, which indicates practical absence of quisinostat in these formulations. The results clearly demonstrate that the drug is only present in the formulation with PPEG and was lost when subjected to dialysis procedure in the absence of the polymer.

## Appendix B

**Table A1:** IC_50_ values of quisinostat–PPEG complexes in A549 NSCLC cells (72 h): IC_50_ values calculated on the basis of complex or polymer concentrations.

Values	PPEG	Complexes (Molar Quisinostat-to-PPEG Ratios)			
		14	38	51	60
IC_50_ (nM)	-	63	84	9	9
95% CI (nM)	-	36–113	46–162	6–12	6–12
R^2^	Non fit	0.94	0.94	0.98	0.97

**Table A2:** IC_50_ values of quisinostat–PPEG complexes in A549 NSCLC cells (72 h): IC_50_ values calculated on the basis of quisinostat concentration.

Values	Complexes (Molar Quisinostat-to-PPEG Ratios)			
	14	38	51	60
IC_50_ (nM)	591	2198	301	364
95% CI (nM range)	340–1067	1211–4220	210–424	255–516
R2	0.94	0.94	0.98	0.97

**Table A3:** IC_50_ values of quisinostat–PPEG complexes in WM115 melanoma cells (72 h): IC_50_ values calculated on the basis of complex or polymer concentrations.

Values	PPEG	Complexes (Molar Quisinostat-to-PPEG Ratios)			
		14	38	51	60
IC_50_ (nM)	-	7.6	5.25	6.67	4.54
95% CI (nM)	-	3.8–14	2.16–11.52	4.67–9.36	2.8–6.8
R^2^	Non fit	0.91	0.91	0.98	0.97

**Table A4:** IC_50_ values of quisinostat–PPEG complexes in WM115 melanoma cells (72 h): IC_50_ values calculated on the basis of quisinostat concentration.

Values	Complexes (Molar Quisinostat-to-PPEG Ratios)			
	14	38	51	60
IC_50_ (nM)	72	136	228	184
95% CI (nM)	36–135	56–299	160–321	117–278
R^2^	0.92	0.92	0.98	0.97
